# High risk feet in subacute rehabilitation facilities: how many are there?

**DOI:** 10.1186/1757-1146-6-S1-O11

**Published:** 2013-05-31

**Authors:** Brenton J Earl, Peter A Lazzarini, Ewan M Kinnear, Petrea L Cornwell

**Affiliations:** 1 Brighton Health Campus & Services, Metro North Hospital and Health Service, Queensland Health, Brisbane, Queensland, 4017, Australia; 2 Department of Podiatry, Metro North Hospital and Health Service, Queensland Health, Brisbane, Queensland, 4032, Australia; 3 Allied Health Research Collaborative, Metro North Hospital and Health Service, Queensland Health, Brisbane, Queensland, 4032, Australia; 4 School of Clinical Sciences, Queensland University of Technology, Brisbane, Queensland, 4059, Australia; 5 Behavioural Basis of Health, Griffith Health Institute, Griffith University, Brisbane, Queensland, 4122, Australia

## Background

Australian subacute rehabilitation facilities face significant challenges from the ageing population with increased burden of chronic disease. High risk foot complications are a negative consequence of many chronic diseases. With the rapid expansion of subacute services, it seems imperative to investigate the prevalence of foot complications in this population. The primary aim of this study was to quantify the high risk foot complication prevalence in a subacute rehabilitation population.

## Methods

Eligible participants were all adults admitted overnight, over two 4 week periods, into a large Australian subacute rehabilitation facility. Consenting participants underwent a short non-invasive foot examination by a podiatrist. The standard Queensland Health High Risk Foot Form collected data on age, sex, co-morbidities and foot complications. Descriptive statistics, logistic regression and odds ratios were used to determine the prevalence of foot complications and associations with explanatory variables.

## Results

Overall, 85 of 97 eligible participants consented; mean age 80(9) and 71% were female. At least one foot complication was present in 56.5% participants; including 21.2% defined as high risk and 11.8% current foot ulcer. A previous diagnosis of neuropathy increased the risk of presenting with a high risk foot by 13-fold (OR 13.504, p = 0.001).

## Conclusion

This study highlights the significance of foot complications in the subacute population. It appears that one in every two patients present with a foot complication and one in eight with a foot ulcer. It is suggested all patients admitted to subacute rehabilitation services should be screened for foot complications.

